# The IFIT3 Protein of Porcine Induces Interferon Signaling and Inhibits the Early Gene Expression of African Swine Fever Virus

**DOI:** 10.3390/v18050566

**Published:** 2026-05-17

**Authors:** Wen-Li Wang, Deng-Wu Han, Xing Yang, Xi-Juan Shi, Ye-Sheng Shen, Shu-Yao Tian, Zhi-Hai Chang, Deng-Ji Zhang, Qiao-Ying Zeng, Shi-Jun Bao, Hai-Xue Zheng, Ruo-Qing Mao

**Affiliations:** 1 College of Veterinary Medicine, Gansu Agricultural University, Lanzhou 730070, China; wangwl0829@126.com (W.-L.W.); shixijuan103@163.com (X.-J.S.); 2 State Key Laboratory of Animal Disease Control and Prevention, College of Veterinary Medicine, Lanzhou University, Lanzhou Veterinary Research Institute, Chinese Academy of Agricultural Sciences, Lanzhou 730046, China; yx73268@163.com (X.Y.); ysshen1997@163.com (Y.-S.S.); tsy05120512@163.com (S.-Y.T.); zhihaichang168@163.com (Z.-H.C.); 3 Gansu Animal Disease Prevention and Control Center, Lanzhou 730070, China; handw6903@163.com (D.-W.H.); zhangdj8203@163.com (D.-J.Z.); 4 African Swine Fever Regional Laboratory of China, China Key Laboratory of Animal Virology of Ministry of Agriculture, Lanzhou Veterinary Research Institute, Chinese Academy of Agricultural Sciences, Lanzhou 730046, China

**Keywords:** ASFV, F334L, IFIT3, type I interferon signaling, adsorption, internalization

## Abstract

African swine fever virus (ASFV) is the causative agent of African swine fever (ASF), a fatal and highly contagious disease, resulting in enormous losses to the global swine industry. No licensed vaccines or effective therapeutics are currently available to control ASFV infection. Interferons (IFNs) serve as key mediators of host antiviral immunity by inducing interferon-stimulated genes (ISGs), but the specific mechanisms by which individual ISGs restrict ASFV replication remain unclear. Interferon-induced protein with tetratricopeptide repeats 3 (IFIT3, also called ISG60) has been shown to exhibit antiviral activity against various viruses, but its role in ASFV infection has not been previously studied. Here, we used porcine alveolar macrophages (PAMs), the primary target cells of ASFV, to investigate IFIT3’s function in ASFV replication. We found that overexpression of IFIT3 inhibited ASFV replication, while its knockdown enhanced viral propagation. Mechanistically, IFIT3 directly blocked ASFV adsorption to host cells, thereby suppressing all subsequent stages of the viral cycle. IFIT3 also specifically interacted with ASFV F334L, an early viral gene product that encodes the small subunit of ribonucleotide reductase, a key enzyme for viral DNA synthesis. Additionally, IFIT3 positively regulated the STAT1/TBK1/IRF3 signaling axis: its overexpression increased phosphorylation of TBK1 and IRF3, as well as the protein level of STAT1, while IFIT3 knockdown attenuated activation of these molecules. Transcriptomic analysis of IFIT3-knockout PAMs revealed significant suppression of innate immune pathways, including type I interferon, JAK-STAT, and RIG-I-like receptor pathways, along with downregulated expression of core antiviral molecules such as ISG15, MX1, and STAT1. Conversely, pathways related to viral adsorption, endocytosis, and cytoskeleton were activated, and pathways involved in protein translation initiation, endoplasmic reticulum stress, and autophagy were dysregulated, creating a favorable intracellular environment for ASFV replication. In conclusion, IFIT3 restricts ASFV replication possibly by inhibiting viral adsorption and promoting innate immune signaling, identifying it as a potential therapeutic target against ASFV. This study’s limitation is its in vitro PAM model; future work will validate IFIT3’s role in vivo and develop targeted inhibitors.

## 1. Introduction

Currently, the global sustainable development of pork production encounters numerous significant challenges [1,2]. African swine fever (ASF) is a highly contagious disease that presents a substantial threat to domestic pigs, exhibiting a mortality rate that can reach 100% [3]. First identified in Kenya in 1921 [4], ASF has since spread from Africa to Europe on three occasions and reached Asia in 2018 [5], resulting in considerable economic losses for the global pig farming industry. As of now, many countries in Europe and Asia continue to experience localized outbreaks of ASF [4]. The African swine fever virus (ASFV) is the sole member of the *Asfarviridae* family [5]. This large double-stranded DNA virus possesses a genome ranging from approximately 170 kb to 193 kb [6]. The ASFV genome encodes over 200 viral proteins [7], yet the functions of more than half of these proteins remain poorly understood. ASFV particles exhibit a five-layer structure, with 68 viral proteins [8] identified as contributors to this formation through mass spectrometry. Phylogenetically, ASFV is classified within the evolutionary branch of *nucleoplasmic* large DNA viruses, sharing a group with poxviruses, iridoviruses, algal DNA viruses, and *pseudoviruses* [9]. Notably, ASFV and poxviruses constitute a distinct lineage, exhibiting numerous shared characteristics in genomic structure, including hairpin loops and terminal reverse repeat sequences, core genomes, and potential replication strategies [10,11].

Interferon-induced protein with tetratricopeptide repeats 3 (IFIT3) is a member of the IFIT protein family, which is localizes to the cytoplasm and has garnered considerable attention due to its notable antiviral properties [12]. The IFIT family comprises four members: IFIT1, IFIT2, IFIT3, and IFIT5, all of which are clustered on porcine chromosome 10q23.31 [13]. While these proteins lack known enzymatic activity, they are characterized by unique tetratricopeptide repeat (TPR). The TPR serves as a structural component of IFIT proteins, consisting of 3 to 16 repetitive and modified tandem sequences, each comprising 34 amino acids. These TPRs are arranged in a helical-angular-helical configuration, facilitating their involvement in protein–protein interactions and various biological processes within cells. The four proteins exhibit conserved structures in their N-terminal regions, which contain the first three TPR domains. However, sequence conservation among the IFIT proteins diminishes toward the C-terminal region, leading to increased structural diversity [14]. IFIT proteins play significant roles in multiple biological processes, including cell proliferation, migration, virus-induced translation initiation, replication, and double-stranded RNA signal transduction. The transcription of the IFIT genes can be rapidly induced by viral infection and interferon (IFN) [15].

IFIT3 is a crucial member of the IFIT family and plays an important role in the innate immune system. It is participates in multiple key signaling pathways, including JAK-STAT, interferon (IFN), and Toll-like receptor (TLR)-mediated immune response pathways [16]. These pathways enhance the host defense mechanism, bolster the immune response, and promote pathogen clearance [17]. Additionally, IFIT3 plays a vital role in pathogen recognition and elimination. Our laboratory previously demonstrated through single-cell sequencing that the expression of IFIT3 markedly upregulated following ASFV infection in porcine alveolar macrophages (PAMs) [18]. Consequently, we hypothesize that the IFIT3 protein may be instrumental in the replication process of ASFV. Herein we aimed to investigate the role of IFIT3 during ASFV replication and elucidate the underlying molecular mechanism. Mass spectrometry confirmed the interaction between IFIT3 and four ASFV proteins. Co-immunoprecipitation further verified the binding of IFIT3 to the ASFV protein F334L. Confocal laser scanning microscopy experiments demonstrated co-localization of IFIT3 and ASFV-F334L. The ASFV F334L gene belongs to the early viral genes and encodes the small subunit pF334L of ribonucleotide reductase. We therefore hypothesize that IFIT3 may affect the early stage of ASFV replication.

## 2. Materials and Methods

### 2.1. Cells and Viruses

HEK-293T (ATCC, CRL-11268), MA104 cells (African Green Monkey Fetal Kidney Epithelial Cells) and PAM cells were cultured in an environment maintained at 37 °C with 5% carbon dioxide, supplemented with 10% fetal bovine serum (FBS) (Gibco, 16140071, Waltham, MA, USA) and 5 mL penicillin/streptomycin/gentamicin (Gibco, 15070063, Waltham, MA, USA), respectively. HEK-293T and MA104 cells (African Green Monkey Fetal Kidney Epithelial Cells cultured in Dulbecco’s Modified Eagle Medium (DMEM) (Invitrogen, 12634010, Waltham, MA, USA) while PAM cells cultured in 1640 medium (Invitrogen, 11875093, Waltham, MA, USA) The 1640 medium was specifically employed for the cultivation of African swine fever virus (ASFV) strains under the same temperature and carbon dioxide conditions. The ASFV-gfp strain was previously preserved in our laboratory [19]. ASFV was diluted in 1640 medium for the inoculation of PAM cells. After two hours, the unadsorbed ASFV was removed, and the cells were cultured in DMEM supplemented with 10% FBS and 1% penicillin/streptomycin/gentamicin. Given that PAMs are primary cells, the packaged IFIT3 overexpression lentiviruses were used to infect PAMs in this study to achieve stable overexpression of IFIT3 in these cells. The lentivirus packaging procedure is described as follows: For lentivirus particle preparation, we employed a three-plasmid system consisting of Plasmid of Slow Virus Packaging Auxiliary X2 (PSPAX2), Plasmid of Mammalian Expression and Viral Envelope G (pMD2.G), and the target plasmid interferon-induced protein with tetratricopeptide repeats 3 (IFIT3). These plasmids were transfected into HEK-293T cells. The supernatant was collected 48 h post-transfection, concentrated using a lentivirus concentration solution (Boaolong, Shanghai, China), and stored at −80 °C.

Porcine alveolar macrophages (PAMs) were prepared in our laboratory. Fresh lungs were isolated from healthy 60-day-old piglets, immediately transported on ice to the cell culture room, and rinsed with PBS supplemented with triple antibiotics before processing in a biological safety cabinet. The trachea was clamped and elevated, and ~500 mL pre-chilled PBS was injected via a 50 mL syringe. After sealing the trachea with hemostatic forceps, the lungs were vigorously massaged for 1–2 min, and the lavage fluid was collected. This lavage was repeated three times to yield ~1 L pooled bronchoalveolar lavage fluid, which was aliquoted into 50 mL centrifuge tubes, balanced, and centrifuged at 1500 rpm for 15 min. The supernatant was discarded, and cells from 5 to 6 tubes were pooled and resuspended in 5–6 mL red blood cell lysis buffer by gentle pipetting. Following 10 min of ice incubation, the lysed PAMs were centrifuged at 1500 rpm for 5 min. The resulting PAM pellet was resuspended in cell freezing medium, aliquoted into cryovials, and stored in liquid nitrogen for subsequent use.

### 2.2. RNA Extraction and RT-qPCR

In this experiment, all cells requiring RNA extraction as specified in the experimental design shall be subjected to RNA extraction after collection. Total RNA was extracted using the Trizol kit (Thermo Fisher, 15596026CN, Waltham, MA, USA) following the manufacturer’s operating procedures. RNA precipitation was washed with 75% ethanol (Dezhou, China), air-dried for 5–10 min, and subsequently dissolved in DEPC-treated water. The RNA concentration and the absence of protein or organic compound contamination were assessed by measuring the OD260/280 ratio using the Nanodrop ND-1000 spectrophotometer (Thermo Fisher). The extracted RNA was reverse transcribed into cDNA using the PrimeScript IV first-strand cDNA synthesis premix reagent (TaKaRa, RR092S, Osaka, Japan). mRNA expression levels were quantified by RT-qPCR using the SYBR qPCR premix reagent (Vazyme, Shanghai, China) on the ABI QuantStudio 5 real-time fluorescence quantitative PCR system in China (Thermo Fisher). The expression level of the target gene was normalized to that of glyceraldehyde-3-phosphate dehydrogenase (GAPDH), which served as the internal reference. The primer sequences utilized in RT-qPCR are presented in Table 1 [20].

### 2.3. Immunofluorescence Staining for Protein Expression Detection

PAM cells were fixed at room temperature using 4% paraformaldehyde for 20–30 min, followed by two washes with Phosphate-Buffered Saline (PBS). Subsequently, the cells were permeabilized with 0.25% Triton X-100 (P0096, Biota Biotechnology, Shanghai, China) for 10 min. The cells were then incubated overnight at 4 °C with the following primary antibodies: rabbit anti-Flag (ab7817, 1:200) and mouse anti-MYC (1:200). After three washes with Phosphate-Buffered Saline PBS, the cells were incubated for 1 h with secondary antibodies: goat anti-mouse Alexa Fluor 594 (ab150083, 1:200) and goat anti-rabbit Alexa Fluor 488 (ab150077, 1:200). Following three additional washes with PBS, the cells were stained with 4′,6-diamidino-2-phenylindole (DAPI) (D3571, 10 μg/mL) at room temperature for 10 min. The slides were stored at 4 °C until further analysis.

### 2.4. Western Blot Analysis and Co-IP

HEK-293T/PAM cells were cultured in 12-well plates or 100 mm culture dishes. Cells were harvested using Radio Immunoprecipitation Assay (RIPA) lysis buffer (Beyotime, Shanghai, China) supplemented with protease inhibitors. Following this, the cells were lysed via ultrasound for 15 min, and the samples were incubated on ice. The cell lysates were centrifuged at 12,000× *g* for 10 min, and the supernatant was collected for immunoprecipitation (IP) treatment. Proteins were separated using a 10% SDS-PAGE gel and subsequently transferred to nitrocellulose membranes (Millipore, St. Louis, MO, USA). For Western blotting, the nitrocellulose membrane was blocked with 5% skimmed milk (BD, Franklin Lakes, NJ, USA) at room temperature for 1 h, followed by three washes with Tris-Buffered Saline with Tween-20 (TBST). The membranes were then incubated overnight at 4 °C with the primary antibody and washed three times with PBST. Finally, the nitrocellulose membrane was incubated with the secondary antibody at room temperature for 2 h and washed three times with Phosphate-Buffered Saline with Tween-20 (PBST).

The target protein was detected following incubation of the nitrocellulose membrane with an Enhanced Chemiluminescence (ECL) luminescent solution. Anti-Flag antibodies (Proteintech, Wuhan, China, diluted 1/300) were employed for detection. To analyze the expression of IFIT3 in pigs, anti-myc antibodies (Proteintech, China, diluted 1/2000) were utilized. The corresponding endogenous proteins were detected using anti-β-actin monoclonal antibodies (Proteintech, China, diluted 1/10,000), anti-STAT1 polyclonal antibodies (Proteintech, China, diluted 1/4000), anti-TBK1 antibodies (Proteintech, China, diluted 1/4000), and anti-IRF3 antibodies (Cell Signaling Technology, Danvers, MA, USA, diluted 1/3000). The phosphorylated forms of each endogenous protein were identified using anti-phospho-STAT1 antibodies (Cell Signaling Technology, USA, diluted 1/3000), anti-phospho-TBK1 antibodies (Affinity, Cincinnati, OH, USA, diluted 1/3000), and anti-phospho-IRF3 antibodies (Cell Signaling Technology, USA, diluted 1/3000). Protein A + G resin magnetic beads (Beyotime, Shanghai, China) were pretreated with lysis buffer. Subsequently, anti-Flag or anti-IFIT3 antibodies were added to the pretreated supernatant and incubated overnight on a shaker at 4 °C. Following this, fully resuspended Beyotime Protein A + G resin magnetic beads were added and incubated on a shaker at 4 °C for 1–3 h. The supernatant was then carefully aspirated by centrifugation at 1000× *g* for 5 min at 4 °C. The precipitate was washed five times with the lysis buffer or Phosphate-Buffered Saline PBS used for protein sample preparation and centrifuged under the same conditions. Finally, the protein sample was prepared for Western blotting analysis.

### 2.5. Eukaryotic Internal Reference High-Throughput Transcriptome Sequencing

In this experiment, PAM cells were transfected with IFIT3 siRNA. After 24 h of transfection, the cells were infected with ASFV at an MOI of 1 and designated as the experimental group. In parallel, PAM cells transfected with IFIT3 siRNA but without ASFV infection served as the control group. Each group was established in triplicate. Transfect IFIT3-SiRNA into adhered PAM cells in large dishes, utilizing freshly isolated PAM cells to ensure experimental accuracy. Three parallel groups were established. Following 24 h transfection with IFIT3-SiRNA, PAM cells were inoculated with ASFV (MOI = 1); the control group (*n* = 3) remained uninoculated. At 24 h post-inoculation, cells were harvested, immediately placed in TRIzol reagent, and rapidly frozen in liquid nitrogen to preserve RNA stability. Total RNA was isolated from each sample using TRIzol reagent (T102096; Sigma-Aldrich, St. Louis, MO, USA) per the manufacturer’s instructions, and RNA quality and concentration were assessed via ultraviolet–visible spectrophotometry (NanoDrop, Agilent, Santa Clara, CA, USA). The transcriptome sequencing of six samples was performed in this study, yielding a total of 41.21 Gb of Clean data. The effective data volume of each sample ranged from 6.66 to 7.0 Gb, with the Q30 ratio distribution between 97.6% and 98.27%. The average GC content was 50.11%. Reads were mapped to the reference genome, the genomic alignment for each sample was determined, resulting in alignment rates from 79.84% to 97.96%. Based on these mapping results, the expression levels of protein-coding genes were analyzed. Differential expressed genes were screened according to the expression levels of protein-coding genes were quantified. one differential comparison group was set up, and a total of 2087 differential expressed genes were identified.

Clean reads were mapped to the designated reference genome using HISAT2, which provided positional information on the reference genome or gene, as well as sequence features specific to the sequencing sample. Utilizing known reference gene sequences and annotation files as a database, the expression abundance of each protein-coding gene in each sample was determined via sequence similarity alignment. The HTSeq-count software v 0.11.2 was used to quantify the number of reads aligned to protein-coding genes in each sample, facilitating the analysis of gene expression levels. The differential expression analysis was performed to identify genes exhibiting differential expression across various samples. Following the identification of differentially expressed genes between the experimental group and the control group, Gene Ontology (GO) functional enrichment and Kyoto Encyclopedia of Genes and Genomes (KEGG) pathway enrichment analyses were performed.

Gene read counts of each sample were normalized using DESeq2software v 1.22.2, employing the Base Mean value to estimate expression levels. Expression differences were calculated, and the significance of these differences was assessed using a negative binomial distribution test (NB). Differentially expressed protein-coding genes were screened according to fold changes and significance test results. Following the identification of differentially expressed genes, Gene Ontology (GO) enrichment analysis was performed to annotate their functions, integrating the GO annotation results. The methodology for GO functional enrichment analysis involved counting the number of differentially expressed genes associated with each GO entry and calculating the significance of their enrichment using the hypergeometric distribution algorithm. This calculation yields a *p*-value indicating the level of enrichment significance, with Fisher’s exact test applied to each term in biological process (BP), cellular component (CC), and molecular function (MF). A lower *p*-value corresponds to greater statistical significance. Candidate genes for subsequent validation and functional research can be selected by combining GO enrichment results with their biological roles.

### 2.6. Proteome Sequencing

Following proteolysis, the peptide segments were analyzed via liquid chromatography–mass spectrometry (LC-MS). Relative protein quantification was performed by comparing the signal intensities of the peptide segments across various samples. The abundance of each peptide segment was directly proportional to its peak area or signal intensity in the mass spectrometer. With reference to the spectral library and mapping the data to proteins, a quantitative analysis of the relative expression levels of proteins in different samples was conducted.

Quantitative proteomics analysis: For the control (Pr-MOCK) group (*n* = 3) and the experimental (Pr-TEST) group (*n* = 3), we collected PAM cells and extracted proteins using RIPA buffer supplemented with protease inhibitors. During the extraction process, we removed the frozen sample and transferred an appropriate volume to a 1.5 mL centrifuge tube. We then added the sample lysate, followed by the phosphatase inhibitor and the protease inhibitor PMSF to achieve a final concentration of 1 mM. Ultrasonic disruption was performed on ice at a power setting of 80 W, with a cycle of 1.0 s on and 1.0 s off, for a total duration of 2 min. The solution was subsequently centrifuged at 12,000× *g* for 10 min at 4 °C. The supernatant was collected and centrifuged again to obtain a clarified supernatant, which constituted the total protein solution of the sample. Following the determination of protein concentration using the Bicinchoninic Acid BCA protein assay, aliquots were prepared and stored at −80 °C for future use.

Sample concentrations were adjusted using the standard curve to confine measurements within a valid range. Total protein was extracted from each sample and separated by 4–12% SDS-PAGE. After enzymatic digestion and peptide desalting, samples were analysed by high-resolution LC-MS/MS. Prior to injection, each sample was mixed with iRT internal standard at a 1:20 volume ratio. Raw MS data were processed in DIA-NN v 2.5.0 for library searching and DIA-based protein quantification. Only proteins with ≥1 unique peptide were retained.

We required no fewer than two valid quantitative values across all samples, and kept proteins with valid values in over 50% of replicates in at least one group. Group-level missing values with >50% valid data were imputed with group means, while remaining blank values were filled using half the global minimum intensity. The resulting data were median-normalised and log_2_-transformed to obtain robust proteins for subsequent analysis.

### 2.7. Statistical Analysis

All experiments in this study were conducted in triplicate. The results presented in the bar chart were analyzed using GraphPad Prism 9 software, with values expressed as the mean ± standard deviation of the three replicates. Statistical significance was analyzed by Student’s *t*-test, and data distribution was evaluated using normality tests. A *p* value of <0.05 was considered indicative of a statistically significant difference.

## 3. Results

### 3.1. IFIT3 Is Induced by AFRICAN Swine Fever

**Figure 1:**
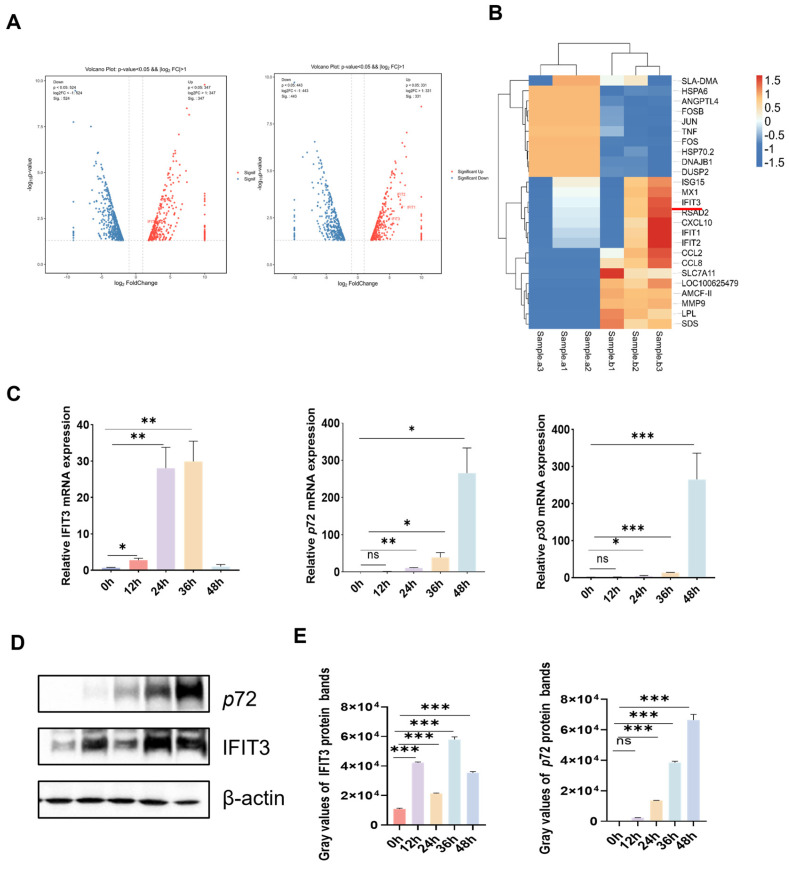
ASFV induced IFIT3 expression. (**A**) Volcano plot showing the global differentially expressed genes in ASFV-infected PAM cells compared with mock PAM cells at 24 h and 36 h. (**B**) Heatmap showing the fold changes in the expression of genes in ASFV-infected PAM cells compared with mock PAM cells at 24 h. The target protein IFIT3 in this experiment is highlighted by the red line. (**C**) The PAM cells were infected with ASFV for 0, 12, 24, 36, or 48 h. The cells were lysed and collected, and the IFIT3, ASFV p72 gene and ASFV p30 gene mRNA levels were then measured by RT-qPCR. All data are reported as means ± SDs. For all experiments, * *p* < 0.05, ** *p* < 0.01, and *** *p* < 0.001 were considered to indicate statistical significance, ns no significance. (**D**) The PAM cells were infected with ASFV (MOI = 1) for 12, 24, and 36 h and were then harvested for Western blot analysis. (**E**) Relative expression levels were quantified using ImageJ software v 1.8.0 and normalized to β-actin.

To investigate the interaction between the virus and host genes during African swine fever virus (ASFV) infection, porcine alveolar macrophage (PAM) cells were infected with ASFV at an MOI of 1. After 24 h of infection, when the majority of the cells had been infected, we conducted RNA-seq transcriptome sequencing analysis [21]. We analyzed significantly upregulated and downregulated proteins using volcano plots and selected the top 50 proteins with the smallest *p*-values for cluster analysis. The results showed that IFIT1, IFIT2, and IFIT3 were all markedly upregulated, among which IFIT3 exhibited the most significant increase, suggesting that IFIT3 may play a critical role during viral infection. The result show that IFIT3 expression was upregulated upon ASFV infection (Figure 1A,B).

Cells were infected with ASFV at an MOI of 1 for 36 h. The relative mRNA levels of IFIT3 were measured by quantitative real-time PCR (qPCR) at 0, 12, 24, 36 and 48 h post-infection. Compared with 0 h, the relative mRNA levels of IFIT3 gradually increased in a time-dependent manner following ASFV infection (Figure 1C).

Consistently, IFIT3 protein levels in PAM cells at 0, 12, 24 and 36 h post-infection also increased in a time-dependent manner, as determined by Western blotting (Figure 1D,E).

### 3.2. IFIT3 Inhibits the Replication of African Swine Fever Virus (ASFV)

**Figure 2:**
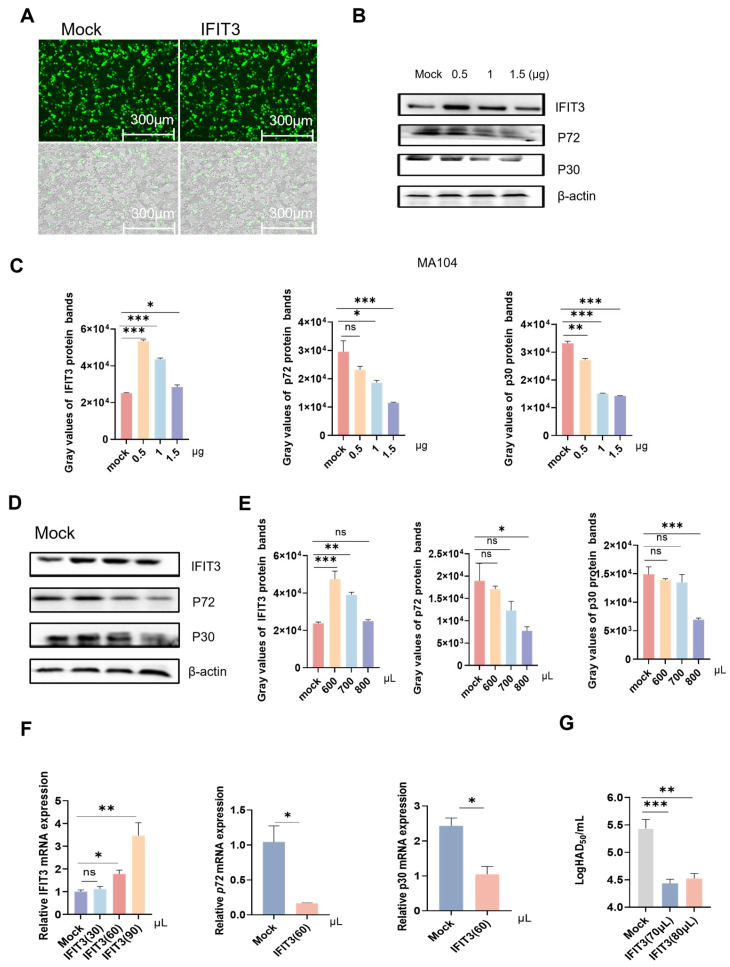
IFIT3 overexpression inhibits ASFV replication. (**A**) Fluorescence map of successful packaging of control empty lentivirus plasmid; Fluorescence map of successful packaging of IFIT3 lentivirus plasmid. (**B**) MA-104 cells were transfected with 0.5, 1, or 1.5 µg of the pCDH-CMV-MCS-EF1-copGFP-T2A-Puro-MYC-pIFIT3, after 28 h of transfection, the cells were infected with ASFV (MOI = 1) for 24 h and were then harvested for Western blot analysis. Relative expression levels in the pCDH-CMV-MCS-EF1-copGFP-T2A-Puro-MYC-pIFIT3, ASFV p72 gene and ASFV p30 gene were determined using ImageJ and normalized to β-actin. (**C**) PAM cells were transfected with Packaged lentiviral plasmid of the pCDH-CMV-MCS-EF1-copGFP-T2A-Puro-MYC-pIFIT3, after 24 h of transfection, the cells were infected with ASFV (MOI = 1) for 24 h and were then harvested for Western blot analysis. Relative expression levels of pIFIT3, ASFV p72 gene and ASFV p30 gene were determined and normalized to β-actin expression using ImageJ. (**D**) PAM cells were transfected with different doses of Packaged lentiviral plasmid of the pCDH-CMV-MCS-EF1-copGFP-T2A-Puro-MYC-pIFIT3, after 24 h of transfection, the cells were infected with ASFV (MOI = 1) for 24 h, The cells were lysed and collected, and the IFIT3, ASFV p72 gene and ASFV p30 gene mRNA levels were then measured by RT-qPCR. (**E**) PAM cells were transfected with empty and IFIT3 lentiviral plasmids, respectively. At 24 h post-transfection, all cells were infected with ASFV (MOI = 1). Supernatants were harvested at 48 hpi to detect the viral titer. Data are presented as the mean ± s.d. of three independent experiments. * *p* < 0.05, ** *p* < 0.01, *** *p* < 0.001. ns, not significant. *p*-values were calculated using a two-tailed unpaired Student’s *t*-tests. (**F**) PAM cells were transfected with the packaged lentiviral plasmid of pCDH-CMV-MCS-EF1-copGFP-T2A-Puro-MYC-pIFIT3. At 24 h post-transfection, cells were challenged with ASFV at an MOI of 1 for 24 h, challenged followed by collection and RT–qPCR quantification of IFIT3, ASFV p72 gene and ASFV p30 gene mRNA levels (**G**) PAM cells were transfected with Packaged lentiviral plasmid of the pCDH-CMV-MCS-EF1-copGFP-T2A-Puro-MYC-pIFIT3, after 24 h of transfection, the cells were infected with ASFV (MOI = 1) for 24 h. Supernatants were harvested at 48 hpi to detect the viral titer. Next, PAM cells were transduced with empty vector or packaged PCDH puro PIFIT3 lentivirus, followed by ASFV infection at an MOI of 1 for 24 h. As shown in Figure 1, during ASFV infection, IFIT3 overexpression reduced the expression of ASFV p72 and p30 proteins in both MA104 and PAM cells (Figure 2B,C). Meanwhile, IFIT3 overexpression markedly decreased ASFV mRNA levels in PAM cells (Figure 2D). Conversely, knockdown of IFIT3 enhanced the expression of ASFV p72 and p30 proteins in PAM cells (Figure 3D,E). Collectively, these results demonstrate that IFIT3 significantly restricts ASFV replication.

To investigate the regulatory role of IFIT3 during ASFV replication, we established both IFIT3 knockdown and overexpression systems. For the knockdown assay, cells transfected with IFIT3-specific small interfering RNAs (siRNAs) served as the experimental group, while those treated with non-targeting negative control siRNAs (NC-siRNAs) were used as the control group. For the overexpression assay, MA104 cells transfected with the IFIT3 overexpression plasmid constituted the experimental group, whereas cells transfected with the empty vector served as the control group. Concurrently, a lentiviral vector encoding porcine IFIT3 was constructed and packaged using a three-plasmid system comprising PSPAX2, PMDG2, and the IFIT3 expression construct (Figure 2A). The resulting IFIT3-overexpressing lentiviruses were concentrated, aliquoted, and stored at −80 °C for subsequent experiments.

MA104 cells were transfected with the IFIT3 plasmid and infected with ASFV at an MOI of 1 at 24 h post-transfection, followed by protein quantification at 24 h post-infection. In parallel, primary porcine alveolar macrophages (PAMs) were transduced with the IFIT3-overexpressing lentiviruses, followed by detection of IFIT3 expression at both the mRNA and protein levels.

**Figure 3:**
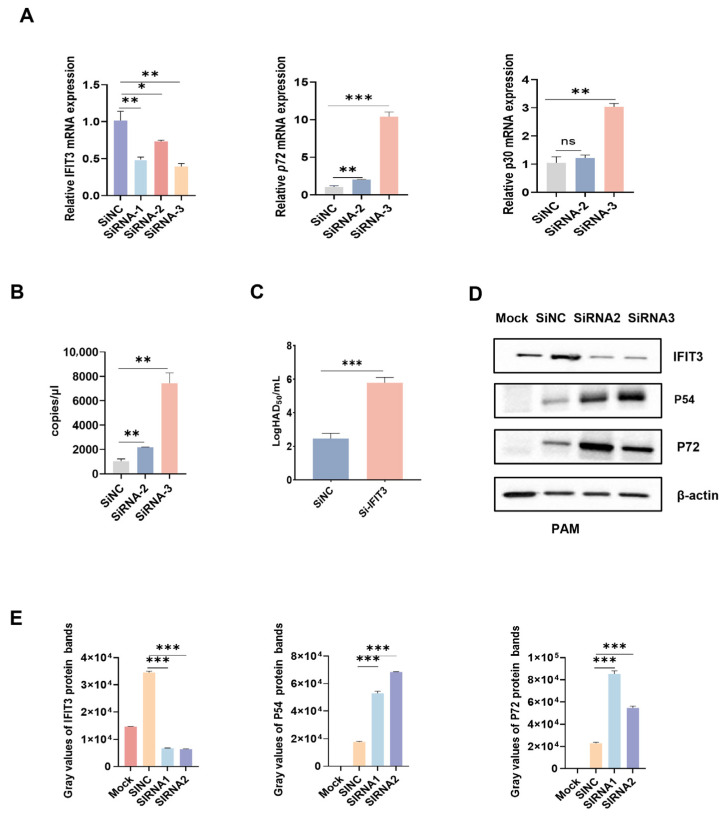
IFIT3 Knockdown Promotes ASFV Replication (**A**) IFIT3 siRNA #1, #2, #3, and negative control siRNA were transfected into PAM cells. After 24 h of transfection, the cells were infected with ASFV (MOI = 1) for 24 h. The cells were lysed and collected, and the IFIT3, ASFV p72 gene and ASFV p30 gene mRNA levels were then measured by RT-qPCR. All data are reported as means ± SDs. For all experiments, * *p* < 0.05, ** *p* < 0.01, and *** *p* < 0.001 were considered to indicate statistical significance. ns, not significant. (**B**) IFIT3 siRNA #1, #2, #3, and negative control siRNA were transfected into PAM cells. After 24 h of transfection, the cells were infected with ASFV (MOI = 1) for 24 h. The cells were lysed and collected, and the ASFV Viral copy numbers were then measured by RT-qPCR. (**C**) Effect of IFIT3 siRNA treatment on ASFV replication. PAM cells were transfected with IFIT3 siRNA, at 24 h post-transfection, all cells were infected with ASFV (MOI = 1). Supernatants were harvested at 48 hpi to detect the viral titer. (**D**,**E**) IFIT3 siRNA #2, #3, and negative control siRNA were transfected into PAM cells. After 24 h of transfection, the cells were infected with ASFV (MOI = 1) for 24 h and then harvested for Western blot analysis. Relative expression levels in the Mock PAM cells, IFIT3 siRNA#2, siRNA#2, and negative control siRNA groups were determined and normalized to β-actin expression using ImageJ.

Compared with the empty vector control group, cells in the PCDH-puro-pIFIT3 group showed markedly reduced ASFV virion production (Figure 2E). In contrast, cells transfected with IFIT3-specific siRNA displayed substantially higher levels of ASFV proteins and relative ASFV mRNA abundance compared with cells transfected with negative control siRNA (NC-siRNA), as determined by immunofluorescence assays (Figure 3A,D,E). Consistently, IFIT3-siRNA-transfected cells also exhibited more numbers of ASFV virions and significantly elevated viral titers (Figure 3B,C).

### 3.3. IFIT3 Deficiency Reduces ASFV DNA Replication at the Early Stage

**Figure 4:**
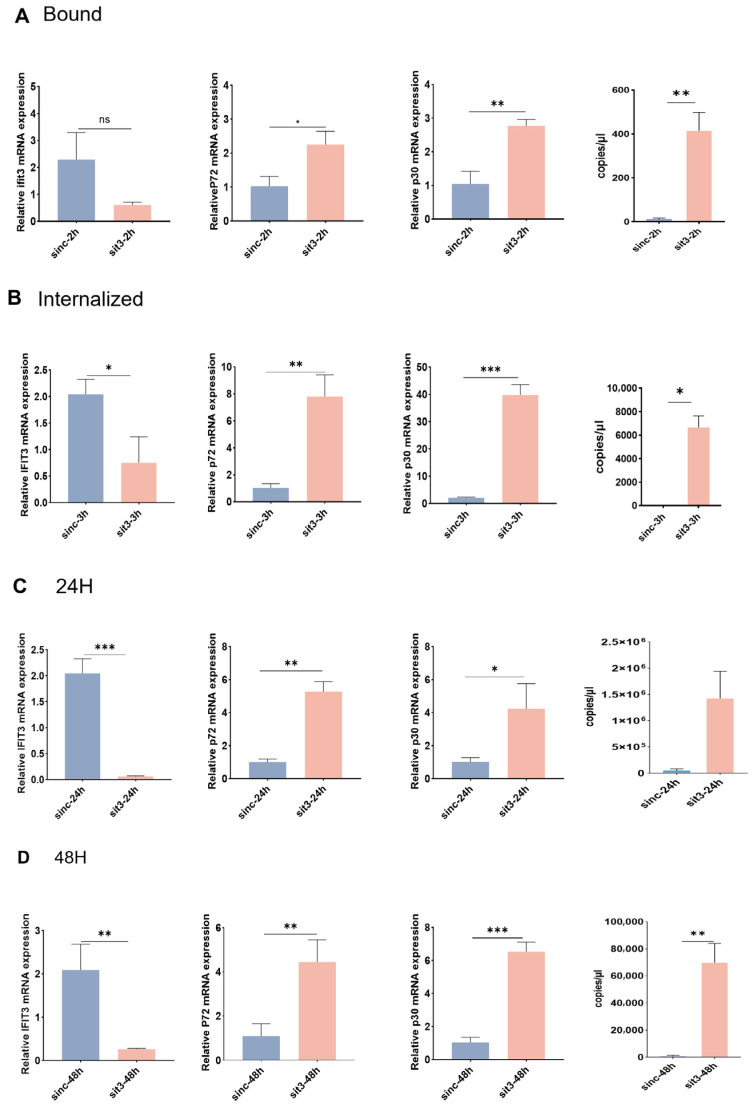

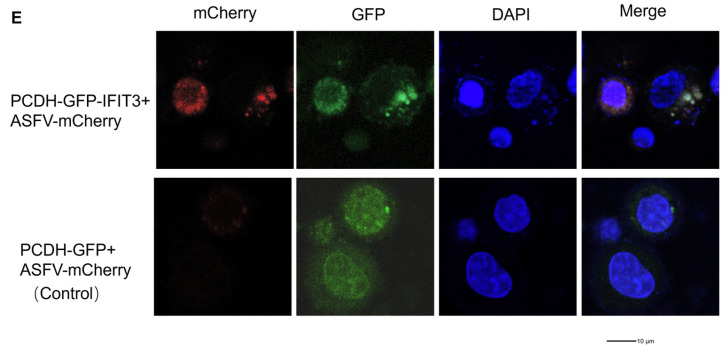
IFIT3 deficiency reduces ASFV replication at the early stage. (**A**,**B**) negative control siRNA and IFIT3 siRNA transfected into PAM cells for 24 h, then incubated with ASFV (MOI of 5) at 4 °C for 1 h (for binding assay, (**A**)), or followed with incubation at 37 °C for 1 h (for internalization assay, (**B**)). The cells were collected, and viral RNA and Viral Copy Numbers was quantified by RT-qPCR. Data were normalized to that of negative control siRNA cells. (**C**,**D**) negative control siRNA and IFIT3 siRNA transfected into PAM cells for 24 h, then both cells were infected with ASFV (MOI of 1). At the indicated time points after infection, the cells were collected, and viral RNA and Viral Copy Numbers was quantified by RT-qPCR. Data were normalized to that of Viral Copy Numbers. * *p* < 0.05, ** *p* < 0.01, *** *p* < 0.001. ns, not significant. *p*-values were calculated using a two-tailed unpaired Student’s *t*-tests. (**E**) The PAMs were transfected with negative control (gNC) and IFIT3 (GFP) (green) for 24 h, cells were infected with ASFV-mCherry (red) (MOI of 5) for 2 h. Confocal microscopy were performed after Nuclei were stained with DAPI (blue). (Scale bar, 10 µm).

To explore the molecular mechanism by which IFIT3 regulates ASFV replication, we first analyzed the influence of IFIT3 on the adsorption and internalization processes of ASFV. IFIT3-siRNA was transfected into PAM cells, with negative control siRNA as the reference. Subsequently, ASFV was used to infect cells in each group, and the viral genomic RNA levels were detected at different time points after infection. The results showed that at 2 h post infection, the content of viral RNA in the IFIT3 knockdown group was significantly higher than that in the control group (Figure 4A). This trend remained consistent at three hours post infection (Figure 4B). However, at 24 h and 48 h post infection, the inhibitory effect of IFIT3 knockdown on viral replication was more significant (Figure 4C,D). The above results suggest that IFIT3 can affect the subsequent replication process of the virus by regulating the adsorption and internalization processes of ASFV.

### 3.4. Bioinformatics Analysis of Host Proteins Interacting with IFIT3 Protein Was Conducted Using Mass Spectrometry

**Figure 5:**
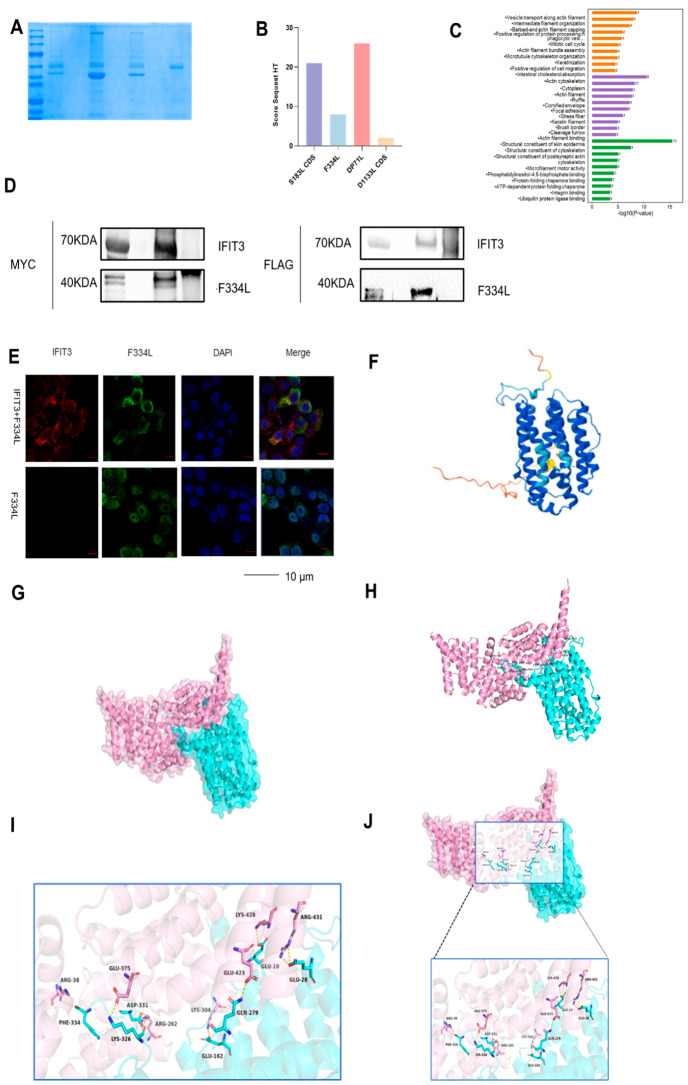
IFIT3 is associated with ASFV-F334L. (**A**) PAM cells were uninfected or infected with ASFV (MOI of 5) for 24 h before Western blot analysis was performed. Immunoprecipitation was performed using an IFIT3 antibody to enrich proteins interacting with IFIT3. (**B**) Screening of ASFV proteins associated with IFIT3 via immunoprecipitation–mass spectrometry. (**C**) The collected samples for LC-MS/MS mass spectrometry analysis. Perform GO enrichment analysis and functional annotation on potential interacting proteins. (**D**) The 293Tcells were cotransfected with F334L-FLAG and IFIT3-MYC. Twenty-four hours after transfection, immunoblotting was performed with the indicated antibodies. (**E**) The 293Tcells were cotransfected with F334L-FLAG (488) (green) and IFIT3-MYC (594) (red). Nuclei were counterstained with DAPI (blue). Twenty-four hours after transfection, confocal microscopy was performed with anti-MYC and anti-FLAG antibodies as indicated. (Scale bar, 10 µm). (**F**) AlphaFold3 Server and PyMOL software v2.5.5 were used to perform molecular structure of IFIT3. (**G**–**I**) AlphaFold3 and PyMOL software v2.5.5 were used to perform molecular docking of IFIT3 with ASFV-F334L, and to generate overall and detailed views of the docking complex. (**J**) AlphaFold3 Server and PyMOL software v2.5.5 were used to perform molecular docking of IFIT3 with ASFV-F334L. Zoomed-in view of the boxed region showing the amino acid composition of the interaction region.

A total of 433 proteins were identified, comprising 429 host proteins and 4 ASFV proteins (Figure 5B). Annotation information for each protein was extracted from databases such as GO, KEGG, InterPro, egg NOG, and Pfam to investigate their functions. During the qualitative analysis, the database search software matched each peptide segment with the background database to determine the coverage index of the peptide segment relative to the complete protein sequence. The coverage for most proteins was below 20%. Subsequently, enrichment analysis and functional annotation were performed on these 433 proteins, further elucidating the functional attributes of host proteins across three dimensions: biological pathways, cellular components, and molecular functions. GO enrichment analysis revealed that the biological processes predominantly associated with the enriched proteins included positive regulation of cell migration, mitotic cell cycle, and receptor aggregation, among others. The cellular components were primarily localized in the cytoplasm, cytosol, actin cytoskeleton, actin fibers, and F-actin cap protein complex. The molecular functions were mainly centered on actin filament binding, protein homodimerization activity, ubiquitin-protein ligase binding, structural composition of the cytoskeleton, and ATP-dependent protein folding chaperones (Figure 5C).

Using a *p*-value threshold of <0.05, KEGG analysis identified several key pathways predominantly associated with cellular processes. These pathways include phagocytosis, regulation of the actin cytoskeleton, gap junction formation, and the generation of neutrophil extracellular traps. Furthermore, it revealed associations with systemic lupus erythematosus, amoebic disease, viral myocarditis, and primary pathways related to immune deficiency and rheumatoid arthritis. To identify proteins interacting with IFIT3, an IP-Mass experiment was conducted, revealing potential ASFV viral proteins that interact with IFIT3. Through mass spectrometry analysis, proteins such as D1133L, S183L, F334L, and DP71L were identified based on factors like Score, viral correlation, and subcellular localization (Figure 5B). The interaction between these viral proteins and IFIT3 may underlie IFIT3’s antiviral activity.

### 3.5. IFIT3 Interact with ASFV F334L

Mass spectrometry identified the interaction between IFIT3 and four ASFV viral proteins. To ascertain which specific protein did not interact with IFIT3, we performed co-immunoprecipitation experiments, which revealed an interaction between IFIT3 and the ASFV viral protein F334L (Figure 5D). Confocal microscopy experiments demonstrated co-localization of IFIT3 and ASFV-F334L (Figure 5E). We predicted the structure of F334L using software. The ASFV gene F334L is classified as an early gene that encodes the small subunit pF334L of ribonucleotide reductase (Figure 5F). F334L has also been implicated as a viral gene involved in nucleotide synthesis and cell cycle regulation. The ASFV genome harbors several genes that contribute to nucleotide and DNA synthesis, including the thymidine kinase gene (K196R), the serine/threonine protein kinase gene (R298L), another thymidine kinase gene (A240L), dUTP nucleotide hydrolase (E165R), and two subunits of ribonucleotide reductase (F334L, F778R) [22,23]. Structural prediction by AlphaFold3 and analysis with PyMOL v2.5.5 revealed a direct interaction between ASFV-F334L and IFIT3 (Figure 5G–I). As shown in the figure, the two proteins form an extensive molecular recognition network via multiple key amino acid residues, including: electrostatic interactions and hydrophobic contacts between GLU28 and ARG431 as well as LYS326 and GLU375, with distances of 3.88 Å and 3.75 Å, respectively; a hydrogen bond and hydrophobic contact between GLN279 andGLU423, with a distance of 3.88 Å; electrostatic and hydrogen-bonding interactions between GLU162 and LYS304, with a distance of 3.70 Å; hydrophobic, electrostatic, and hydrogen-bonding interactions between GLU10 andLYS428, ASP331 and ARG262, as well as PHE334 andARG38, with distances of 3.50 Å, 3.21 Å, and 3.84 Å, respectively (Figure 5J).

### 3.6. Eukaryotic Referenced Transcriptome Sequencing and Data Analysis

We completed sequencing of a total of six samples using reference transcriptome methods, yielding 40.17 Gb of clean data. The effective data volume for each sample varied between 6.1 and 6.99 Gb, with Q30 base distributions ranging from 97.09% to 97.39%. The average GC content was measured at 53.28%. By aligning the reads to the reference genome, we obtained the genomic alignment status for each sample, revealing alignment rates between 95.74% and 96.49%. Utilizing the comparison results, we analyzed the expression levels of protein-coding genes. Differential screening was performed based on the expression levels of these genes across different samples. A total of one differential group was established, which included 97 detected differential genes (as shown in Table 2).

**Figure 6:**
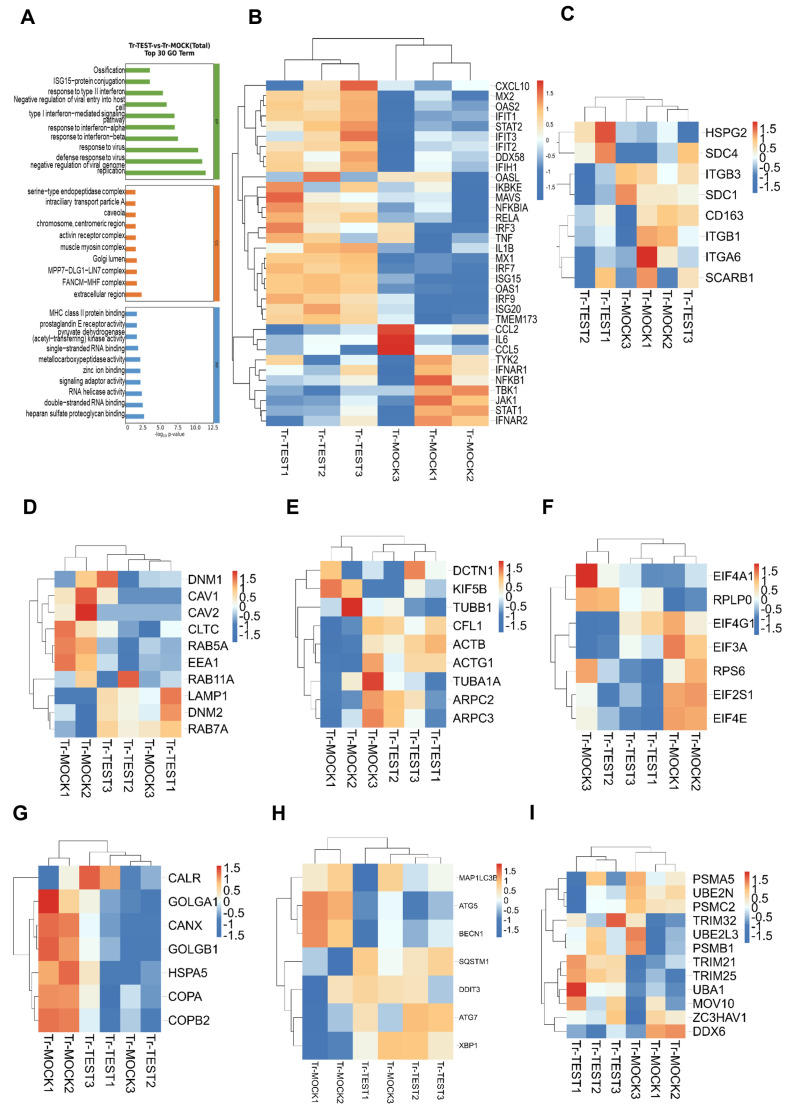
Bioinformatics analysis of transcriptomic data from ASFV-infected cells after IFIT3 knockdown (**A**) Transcriptome-based combined enrichment analysis of innate immune-related pathways. (**B**) Downregulation of core antiviral molecules upon IFIT3 knockdown. (**C**) Upregulation of ASFV adsorption receptors CD163, ITGB1 and SCARB1. (**D**) Upregulation of endocytosis-related factors. (**E**) Upregulation of cytoskeleton molecules. (**F**) Altered expression of translation initiation factors (EIF4E, EIF2S1). (**G**) Significant changes in ER-related molecules (HSPA5, CANX). (**H**) Significant changes in autophagy-related proteins (MAP1LC3B, SQSTM1). (**I**) Proteins are mainly enriched in protein ubiquitination, RNA stability, and translation regulatory pathways (e.g., TRIM25, UBE2N, MOV10).

We identified differentially expressed genes across various samples using DESeq2. Following this identification, we performed Gene Ontology (GO) functional significance and Kyoto Encyclopedia of Genes and Genomes (KEGG) pathway significance analyses. Compared with the control group, the experimental group exhibited 40 significantly upregulated genes and 57 significantly downregulated genes. The upregulated genes include ISG15, ISG12, TCEA3, SRPX, BMP8A, and MUC20, while the downregulated genes include PDK4, CPA2, HEMGN, TPBG, LRRD1, and HRK. The relatively highly expressed protein-coding genes are LOC110258046, HRK, CPA2, PRICKLE2, and LRRD1, among others. In contrast, the relatively low-expressed protein-coding genes consist of MUC20, CCDC103, LOC106507527, SRPX, ISG15, ISG12, and TCEA3 (as shown in Figure 6C). GO enrichment analysis was conducted on differentially expressed genes (DEGs) in PAM cells infected with ASFV compared to those not infected after IFIT3 knockdown, to predict their biological functions. These results indicate that IFIT3 deficiency markedly reshapes the transcriptional and proteomic profiles of PAM cells, and this regulatory effect is further enhanced upon ASFV infection.

Combined enrichment analysis demonstrated that differentially expressed genes (DEGs) and proteins were predominantly enriched in pathways associated with innate immune regulation, viral entry and endocytosis, protein translation and intracellular membrane trafficking, and cytoskeleton organization—all of which are highly consistent with the phenotypic observation that IFIT3 knockout (IFIT3-KO) promotes ASFV adsorption and replication in PAM cells.

Notably, innate immune-related signaling pathways, including the type I interferon (IFN) signaling pathway, JAK-STAT signaling pathway, and RIG-I-like receptor (RLR) signaling pathway (Figure 6A), were significantly repressed in IFIT3-KO PAM cells. This suppression was accompanied by marked downregulation of both transcript and protein levels of core antiviral molecules, including ISG15, MX1, OAS1, IFIT1, STAT1, and IRF7 (Figure 6B). In contrast, pathways governing viral adsorption, endocytosis, and cytoskeleton dynamics—specifically clathrin-dependent endocytosis, actin cytoskeleton assembly, and cell adhesion—were significantly upregulated. Consistent with this activation, the transcript and protein levels of ASFV adsorption receptors CD163, ITGB1, SCARB1 (Figure 6C); endocytosis-related factors CLTC, CAV1, RAB5A (Figure 6D); and cytoskeleton proteins ACTB, ARPC2 (Figure 6E) were substantially increased.

Furthermore, pathways involved in translation initiation, endoplasmic reticulum (ER) stress (unfolded protein response, UPR), and autophagy were profoundly dysregulated in IFIT3-KO cells. Specifically, the expression levels of translation initiation factors EIF4E, EIF2S1 (Figure 6F); ER-associated molecules HSPA5, CANX (Figure 6G) and autophagy-related proteins MAP1LC3B, SQSTM1 (Figure 6H) were significantly altered. Collectively, these changes establish a permissive intracellular microenvironment that facilitates ASFV replication.

Integrated transcriptomic and proteomic analysis revealed 128 differential molecules with consistent mRNA-protein expression trends (synchronous upregulation or downregulation of mRNA and protein), which were primarily involved in pathways related to innate immune regulation and viral replication—suggesting these molecules may be transcriptionally regulated by IFIT3. Meanwhile, 47 differential molecules with inconsistent mRNA-protein expression trends were identified, and these were predominantly enriched in pathways of protein ubiquitination, RNA stability, and translation regulation (e.g., TRIM25, UBE2N, MOV10; Figure 6I), indicating IFIT3 may regulate ASFV infection via post-transcriptional mechanisms. Together, these findings demonstrate that IFIT3 knockout promotes ASFV adsorption and replication in PAM cells by suppressing innate immune responses, activating viral entry-related pathways, and remodeling the intracellular replication microenvironment.

### 3.7. Pro DIA-Based Quantitative Proteomics Sequencing and Bioinformatics Analysis

**Figure 7:**
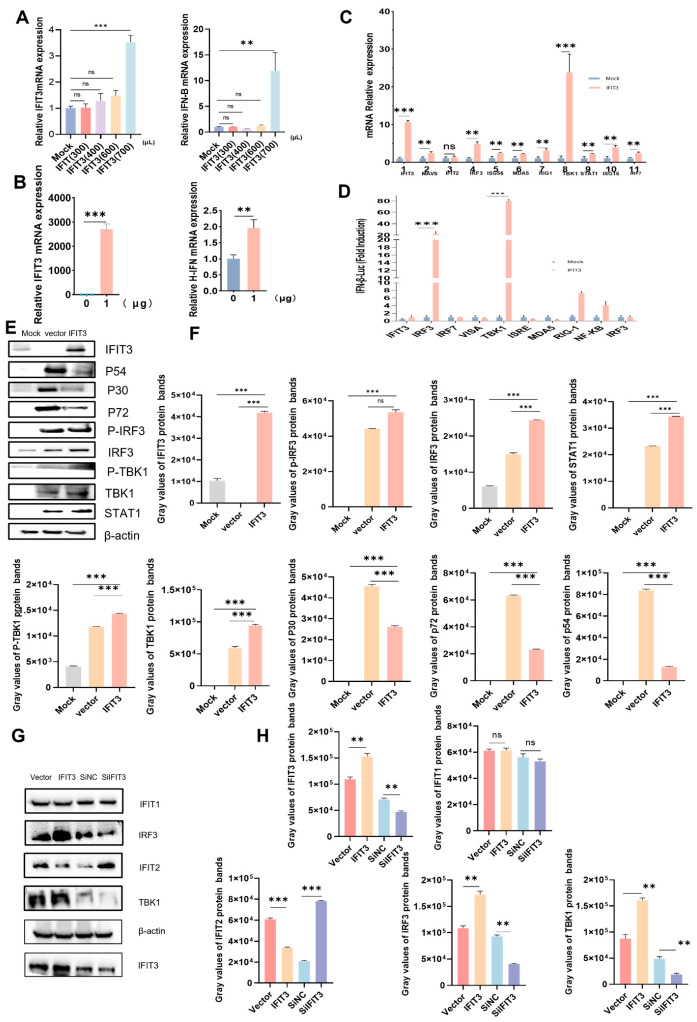
IFIT3 Activates the Type I IFN Signaling Pathway. (**A**) PAM cells were transfected with indicted indicated dose of packaged IFIT3 lentivirus for 24 h. Cells were then harvested, and IFN-β mRNA levels were quantified by RT-qPCR. (**B**) HEK-293T cells were transfected with 0, 1 µg of PCDH-puro-pIFIT3 plasmid for 30 h. Cells were harvested, and IFN-β mRNA levels were analyzed by RT-qPCR. (**C**) PAM cells were transfected with indicated dose of packaged IFIT3 lentivirus, mRNA levels of ISG56, ISG15, MDA5, MAVS, TBK1, RIG-I, NF-κB, IRF3, STAT1, IRF7, and IFIT2 were determined by RT-qPCR. (**D**) HEK-293T cells were co-transfected with 1.5 µg of PCDH-puro-PIFIT3 expression plasmid, together with IFN-β luciferase reporter and pRL-TK internal control plasmids. At 30 h post-infection, cells were lysed and subjected to a dual-luciferase reporter assay. All data are presented as mean ± SD. Statistical significance was determined with ** *p* < 0.01, and *** *p* < 0.001. ns indicates a non-significant difference. (**E**) PAM cells were transfected with PCDH Puro vector, PCDH puro pIFIT3. After 30 h, cells were harvested and analyzed by Western blotting using antibodies against IRF3, p IRF3, STAT1, p STAT1, TBK1, p TBK1, and IFIT3. (**F**) Protein levels of IRF3, p IRF3, and STAT1 were quantified using ImageJ. Relative levels of p STAT1, TBK1, and p TBK1 were normalized to GAPDH. (**G**) PAM cells were transfected with PCDH-Puro-vector, PCDH-puro-PIFIT3, IFIT3 siRNA, or negative control siRNA. After 30 h, cells were harvested and analyzed by Western blotting using antibodies against IRF3, TBK1, IFIT2, IFIT1 and IFIT3. (**H**) Protein levels of indicated were quantified using ImageJ. Both were normalized to GAPDH.

Joint enrichment analysis revealed that differentially expressed genes and proteins were markedly enriched in innate immune signaling pathways, particularly the type I interferon, JAK-STAT, and RIG-I-like receptor pathways. We next experimentally validated these key pathways. Using a dose–response assay, we assessed the effect of IFIT3 on IFN-β promoter activity in HEK-293T and PAM cells. IFIT3 strongly enhanced both IFN-β promoter activity and IFN-β mRNA expression (Figure 7A,B,D).

We then examined the mRNA levels of a panel of antiviral genes, including ISG56, ISG15, MDA5, MAVS, TBK1, RIG-I, NF-κB, IRF3, STAT1, IRF7, and IFIT2, in PAM cells transduced with escalating doses of IFIT3-overexpressing lentivirus. RT-qPCR analysis verified that IFIT3 acts as a potent interferon-stimulated gene (ISG) that is robustly upregulated (Figure 7C). Mechanistically, overexpression of IFIT3 in both PAM and HEK-293T cells elevated the total protein levels and phosphorylation of STAT1, TBK1, and IRF3 (Figure 7E,F). Conversely, IFIT3 knockdown markedly impaired their activation (Figure 7G,H).

### 3.8. Proteomic High-Throughput Sequencing and Data Analysis

**Figure 8:**
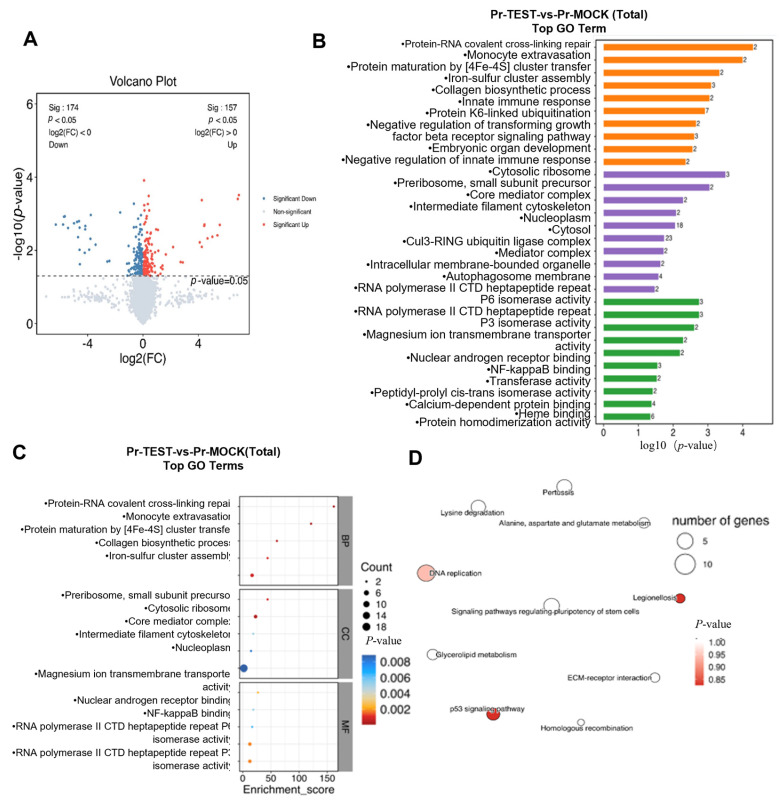
Bioinformatics analysis of Proteomic data from ASFV-infected cells after IFIT3 knockdown. (**A**) In the volcano plot, the x-axis represents log2 (fold change) (log2FC), where values further from the zero indicate greater differential expression. Points on the right denote upregulated proteins, while those on the left indicate downregulated proteins. The y-axis represents −log10 (*p*-value), with values further from the zero reflecting more significant differences. Red and blue dots represent upregulated and downregulated proteins, respectively; the deeper the color, the more significant the differential expression. Gray dots indicate proteins with a *p*-value ≥ 0.05. (**B**) Differential Protein GO Enrichment Analysis. (**C**) The most significantly downregulated pathways identified in the KEGG analysis. (**D**) the pathways that ranked relatively high in the GO analysis.

Following LC-MS/MS detection and database analysis of the samples, a total of 6719 proteins and 54,805 peptide segments were identified. The control group (Pr-MOCK) consisted of 3 samples, the experimental group (Pr-TEST) also included 3 samples. The screening criteria were established as follows: FC ≥ 1.2 or FC ≤ 1/1.2 with *p* < 0.05. In the comparison between Pr-TEST and Pr-MOCK, 89 proteins were found to be upregulated, and 90 proteins were downregulated, resulting in a total of 179 differentially expressed proteins. The *p*-value and fold change (FC) of these differential proteins were visualized using a volcano plot, which clearly delineates the significantly upregulated proteins (upper right) and downregulated proteins (upper left) (see Figure 8A). Subsequently, pathway analyses were performed on all differentially expressed proteins (Total), as well as on the upregulated (Up) and downregulated (Down) subsets of differentially expressed proteins, respectively. For the identified proteins, annotation information was extracted from databases such as GO, KEGG, InterPro, eggNOG, and Pfam to investigate protein functions. According to the GO database, 6437 proteins were detected, with their primary functions predominantly located in the cytoplasm, cytosol, nucleus, nucleoplasm, and ATP binding (see Figure 8B). The KEGG database identified 3654 proteins, with metabolic pathways ranking notably high among their main functions. Specific pathways included neurodegeneration—multiple diseases, amyotrophic lateral sclerosis, Alzheimer’s disease, and pathways in cancer. The egg NOG database revealed 2602 proteins, whose primary functions were largely associated with posttranslational modification, protein turnover, and chaperones, as well as translation, ribosomal structure and biogenesis, transcription, and replication, recombination, and repair. Qualitative and quantitative data from these databases were collected. Following quality assessment and preprocessing, expression level analysis and functional analysis were conducted separately. Functional annotation analysis of the identified proteins was performed using multiple common databases. For the data from the differential comparison group, volcano plots, expression pattern clustering heat maps, and Venn analysis were generated. The differentially expressed proteins obtained through screening underwent three analyses: GO analysis, pathway analysis, and interaction analysis. Additionally, based on data availability, relevant or intriguing aspects were examined, key proteins and their functions or pathways were selected, and subsequent key research and verification directions were pursued.

The enrichment pathway network diagram illustrates the similarities and overlaps among pathways by performing correlation analyses on the ten functional items/pathways exhibiting the largest absolute values of up-regulation or down-regulation of NES in the GSEA analysis results. The most significantly downregulated pathways identified in the KEGG analysis primarily pertain to DNA replication, the P53 signaling pathway, lysine degradation, signaling pathways that regulate stem cell pluripotency, alanine, aspartic acid, and glutamic acid metabolism, as well as ECM receptor interactions (see Figure 8C). Conversely, the top ten upregulated pathways predominantly involve the metabolism of exogenous substances by cytochrome P450, the metabolism of amino sugars and nucleotide sugars, the metabolism of allergens by cytochrome P450, the synthesis and secretion of cortisol, and the biosynthesis of nucleotide sugars. In the GO pathway analysis, the most significantly upregulated pathways are primarily associated with the positive regulation of the classical Wnt signaling pathway, 3-phosphoinositol-dependent protein kinase activity, DNA-dependent protein kinase activity, and the positive regulation of cell migration. Additionally, the pathways that ranked relatively high in the GO analysis focus on the activity of transcriptional co-activators, the positive regulation of classical NF-κB signal transduction, protein homopolymerization, phospholipid binding, phagocytosis, mitochondrial electron transport from NADH to ubiquinone, DNA damage responses, and C-polyubiquitin modifier-dependent protein binding (see Figure 8D).

## 4. Discussion

ASFV is the primary pathogenic agent responsible for mortality in the pig farming industry [24]. As a large DNA virus with a complex structure, the replication biology of ASFV remains inadequately understood, hindering deeper insights into its pathogenic mechanisms.

In addition to its potential direct antiviral activity via mediating translation inhibition [25], IFIT3 may act as a molecular scaffold linking MAVS and TBK1 [26,27], thereby contributing to RIG-I signaling and exerting indirect antiviral effects [28]. Notably, a recent transcriptomic analysis indicated that IFIT3 induction was more pronounced in response to transmissible gastroenteritis virus and PDCoV than to porcine epidemic diarrhea virus (PEDV), supporting the important involvement of IFIT3 in the interferon-mediated antiviral response. Nevertheless, the specific impact of IFIT3 on cells infected with ASFV remains uncertain.

Here, we further validated the interplay between ASFV replication and the host immune response. Our results reveal that knockdown of pIFIT3 enhances ASFV proliferation in porcine alveolar macrophages I, whereas overexpression of IFIT3 suppresses viral replication. Our preliminary results suggest that this inhibitory effect may target the early stage of viral infection, specifically viral adsorption, and further in-depth investigations will be required to validate this conclusion. Collectively, these findings corroborate and extend our previous integrated transcriptomic and proteomic analyses, establishing a robust phenotypic basis for dissecting the underlying molecular mechanisms.

Viral adsorption represents a critical rate-limiting step during ASFV infection, relying on the specific recognition and binding of viral envelope proteins to host cell surface receptors, which allows the virus to breach the host’s first-line defense and initiate infection. In this study, we demonstrate for the first time that IFIT3 directly regulates this early entry process, revealing a novel functional dimension of IFIT family proteins in the host’s early antiviral defense machinery. Whereas most previous studies have focused on the inhibitory roles of IFITs against late infection stages, including viral transcription, translation, and particle assembly, our targeted analyses show that IFIT3 directly modulates viral adsorption—the initial event of infection. Collectively, these findings significantly broaden the known antiviral spectrum of the IFIT family and provide new mechanistic insights into the multifaceted host innate immune defense.

Here, we demonstrate for the first time that IFIT3 directly interacts with the F334L protein encoded by African swine fever virus (ASFV) and colocalizes with it in cells, providing critical experimental evidence to decipher the molecular mechanism underlying IFIT3-mediated antiviral activity. As a core functional protein of ASFV, F334L is well-documented to be involved in multiple key pathogenic processes, including virion assembly and host immune evasion. Its interaction network with host factors serves as a fundamental molecular basis for efficient viral infection and pathogenesis.

The direct binding between IFIT3 and F334L is proposed to exert antiviral effects through two parallel pathways. First, IFIT3 may occupy the key functional domains of F334L, thereby competitively abrogating its interactions with host or viral partner proteins and subsequently suppressing virion assembly and release. Second, IFIT3 can recruit ubiquitination regulators (e.g., TRIM25 and UBE2N) enriched in our proteomic dataset, mediating the ubiquitination of F334L and its subsequent degradation via the proteasomal pathway, which in turn reduces the stability of this core viral protein.

The identification of this IFIT3-F334L interaction uncovers a novel antiviral mode of action for IFIT3, whereby it directly targets a viral protein. This finding addresses the current research gap that most studies have focused on the role of IFITs in regulating host signaling pathways [29], and provides a new perspective for further unraveling the molecular mechanisms by which host innate immunity defends against ASFV infection.

Transcriptomic analysis combined with functional cellular assays suggests that IFIT3 may promote the expression and phosphorylation of STAT1, TBK1, and IRF3—core components of the type I interferon (IFN-I) signaling pathway. These changes could contribute to enhanced the host innate immune response against African swine fever virus (ASFV). As a pivotal serine/threonine kinase in innate immunity; TBK1phosphorylation has been demonstrated to activate the downstream transcription factor IRF3 thereby facilitating IFN-I transcription. Phosphorylated STAT1 participates in regulating the expression of interferon-stimulated genes (ISGs), which may collectively constitute an antiviral network that contributes to limiting viral replication and spread.

Here we show that overexpression of IFIT3 markedly elevates the phosphorylation levels of TBK1 and IRF3, as well as the protein abundance of STAT1, whereas IFIT3 knockdown attenuates the activation of these molecules. These results establish IFIT3 as a positive regulator of the STAT1/TBK1/IRF3 axis involved in modulating host innate immune responses. This study provides new insights into this mechanism during DNA virus (ASFV) infection, supporting the functional conservation of IFIT3 in innate immune regulation and laying experimental groundwork for understanding its broad-spectrum antiviral activity.

Proteomic analysis showed that IFIT3 overexpression significantly altered the expression of autophagy-related molecules (MAP1LC3B, SQSTM1) and endoplasmic reticulum stress-related molecules (HSPA5, CANX). These changes indicate that IFIT3 modulates autophagy and ER stress, and we propose this contributes to its ability to limit ASFV replication by stabilizing intracellular homeostasis.

Collectively, IFIT3 constrains ASFV infection via combined transcriptional and post-transcriptional mechanisms, acting at multiple viral replication cycle stages including adsorption, innate immune signaling modulation, intracellular replication, and virion release. Its primary antiviral effect is exerted by blocking ASFV adsorption at the early infection stage.

These findings define a new antiviral role of IFIT3 in ASFV infection, highlight host–virus interplay, and provide potential molecular targets for developing host-directed strategies to control African swine fever.

## 5. Conclusions

**Figure 9:**
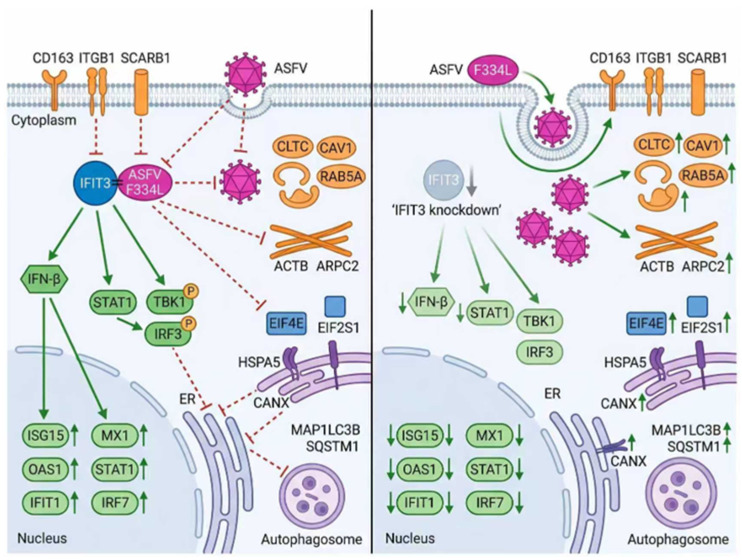
Schematic diagram of IFIT3 negatively regulates ASFV replication. IFIT3 overexpression restricts ASFV replication, while its knockdown facilitates viral propagation. IFIT3 interacts and colocalizes with ASFV F334L protein to block viral adsorption., Transcriptomic profiling of IFIT3-knockout PAM cells revealed suppression of innate immune signaling and decreased expression of host antiviral factors, Loss of IFIT3 also induced the activation of viral adsorption, endocytosis and cytoskeleton-related pathways, together with dysregulated translation initiation, ER stress and autophagy processes which collectively favour ASFV replication). Functional validation demonstrated thatIFIT3 upregulates IFN-β production and activates the TBK1-IRF3- STAT1 signalling cascade, whereas IFIT3 depletion suppresses the activation of this core antiviral axis. The upward arrow represents upregulation. The downward arrow represents downregulation.

In conclusion, IFIT3 overexpression inhibits ASFV replication, while its knockdown promotes viral replication. IFIT3 interacts and colocalizes with ASFV F334L and suppresses ASFV adsorption. Transcriptomic analysis of IFIT3-knockout PAM cells revealed significant suppression of innate immune pathways (type I interferon, JAK-STAT, RIG-I-like receptor) and downregulation of core antiviral molecules (e.g., ISG15, MX1, STAT1), alongside activation of viral adsorption, endocytosis, and cytoskeleton-related pathways and upregulation of their associated molecules. Additionally, dysregulation of protein translation initiation, ER stress, and autophagy pathways creates a favorable intracellular environment for ASFV replication. Validation experiments confirmed that IFIT3 overexpression enhances IFN-β production and activates STAT1, TBK1, and IRF3, while IFIT3 knockdown impairs their activation (Figure 9).

## Figures and Tables

**Table 1:** Primer sequences used in this study.

Primer Name	Primer Sequence
IFIT3-F	GCCATTGAGTTGAGCCCTGAC
IFIT3-R	CACTGCGGAGGACATCTGTTTG
ASFV-p72-F	GCGCTCTGGATTAAGTTGCG
ASFV-p72-R	ATATTGCGTCTACTGGGGCG
Porcine GAPDH-F	ACATGGCCTCCAAGGAGTAAGA
Porcine GAPDH-R	GATCGAGTTGGGGCTGTGACT
ASFV-p30-F	CTCCGATGAGGGCTCTTGCT
ASFV-p30-R	AGACGGAATCCTCAGCATCTTC
IFIT3-Sus-siRNA-1	GAGGAUUAAUUCACAAGAUTT
IFIT3-Sus-siRNA-1	CUUGUGAAUUAAUCCUCTTAU
IFIT3-Sus-siRNA-2	CUGCCCUGGAAUACUUACATT
IFIT3-Sus-siRNA-2	UGJAAGUAUUCCAGGGCAGTT
IFIT3-Sus-siRNA-3	CAGCCCAACAGUCUUUAGATT
IFIT3-Sus-siRNA-3	JAAAGACUGUUGGGCUGTTUC

**Table 2:** List of Preprocessing Results for Sequencing Data Quality.

Sample	Raw Reads (M)	Raw Bases (G)	Clean Reads (M)	Clean Bases (G)	Valid Bases (%)	Q30 (%)	GC (%)
Tr-MOCK1	21.85	6.47	21.47	6.36	98.27	97.15	52.20
Tr-MOCK2	23.96	7.08	23.51	6.95	98.09	97.22	52.61
Tr-MOCK3	20.88	6.20	20.57	6.10	98.52	97.09	53.74
Tr-TEST1	24.07	7.11	23.58	6.96	97.96	97.27	53.99
Tr-TEST2	23.44	6.93	23.01	6.81	98.18	97.27	53.75
Tr-TEST3	24.05	7.12	23.62	6.99	98.22	97.39	53.41

## Data Availability

All the data generated or analyzed during the current study are included in this article.
